# Cotton-Seed Cakes1Read before the twenty-seventh annual meeting of the United States Veterinary Medical Association, at Chicago, September 17th, 1890.

**Published:** 1890-10

**Authors:** J. C. Meyers


### COTTON-SEED CAKES.[1]

By J. C. Meyers, Sr.

The utility attributed to this article as a food supply for domestic animals is, according to my estimation, worthy of consideration. The acknowledged good results obtained, both in hygienic and nutritive processes, by feeding oil cake meal, the residue from flaxseed, seems to have paved the way for the consumption of cotton-seed cake meal. The few accounts I have read as to its efficacy as an aliment were very favorable, but my

[1] Read before the twenty-seventh annual meeting of the United States Veterinary Medical Association, at Chicago, September 17th, 1890.

first and thus far only acquaintance with it, appeared to me of sufficient importance to demand more than a superficial notice. The reason for the above opinion is based on the suspicion of its having caused a disease, with fatal termination, of twenty or more oxen in a Kentucky distillery in May, 1889.

On the 12th of said month, I was requested to inspect the stock in question, and if possible ascertain the nature and cause of the disease, and to facilitate matters, make a few post-mortem examinations. I found 100 or more oxen under one roof, all of which, with a few exceptions, were in fair condition. The building was on elevated ground and well ventilated. The cadavers, which I expected to examine, had been cremated, as had all the others, which died before, it having been forgotten to give contra orders.

The general impression was that the malady was abating, both in number and force, as there were but three sick animals, two of which were on a fair way to recovery; these would not admit of a close examination. They walked about in their pens eating some hay occasionally and drinking water, but paid no attention to mixed food of still swill and cotton seed-cake meal; one even attempted to ruminate several times. The alimentary deposits were soft and of a yellowish-red color. The third steer, sick about thirty-six hours, showed symptoms for the worse; he wore an anxious look, would not move voluntarily, lifted and shifted his feet quite often, shivered almost continually in the muscular region of the extremities. Respiration, 100; temperature, 104°; circulation, 80. White foam dribbled from nose and mouth incessantly. He drank water greedily, but refused all nourishment, had a fit of vertigo, but did not come to a fall. Evacuations from the bowels were mushy, thoroughly mixed with blood, and turned black in fifteen or twenty minutes.

The foreman of the stable stated that the majority of the animals which died showed about the same symptoms as this one.

The first signs of their being sick was drowsiness, salivation, refusal of liquid food, desire for clear water, and evacuations of soft reddish manure. As the disease advanced, a dizziness would very often bring them to a fall, and when down, a tremor of the extremities was present. They then would get up, and move about again; but upon the third attack generally remained down until they expired, several hours after. The disease lasted from two to three days.

Permission to kill this steer was granted. When walking to the place of destruction, a distance of about sixty feet, the animal was attacked with such a dyspnœa that he stopped suddenly, stretched head and neck, protruded the tongue, straddled his legs to prevent his falling, and coughed up some foam, which seemed to relieve him, so that he was able to finish his trip. A single stroke from a broad-headed hammer felled him, after which he was depleted in butcher's manner.

The blood looked like arterial blood, and coagulated in a very short time; the subcutaneous vessels yielded a darker colored fluid. The muscles had a natural aspect. Nothing abnormal was visible on the surface of the alimentary tract. The rumen contained a considerable quantity of partially liquid food, the inner lining was softer than usual; the other three stomachs contained comparatively little food.

The first two and the larger part of the third division of the small intestines were normal. The last fourth of the ileum and the whole tract of the large intestines contained a bloody, mushy, almost odorless mixture. From the last fourth of the ileum to the terminus of the alimentary canal the mucous membrane was stained with blood, which could be washed off, save in the cæcum and first half of the colon, where this sanguineous abnormity penetrated the mucosa and muscular coat to such a depth that it might be termed a bloody infiltration.

Spleen and liver normal, as were also the kidneys. Gall-bladder filled with green-colored bile of molasses consistency.

Water-bladder contained a yellowish translucent urine. Lungs and heart were healthy, except that the bronchial tube and trachea were occupied by a considerable amount of white foam.

The brain was not damaged in the least by the death blow, unless a few congested vessels seen in the arachnoida were the result of it. Whether it was softer than normal cannot be maintained. As there was nothing unusual in the animal's locomotion an examination of the spinal cord was not made, there being little hope that its exposition would lead to any important disclosures. The foreman remarked that in all the cadavers he opened he found nothing that attracted his attention, except white foam in the respiratory apparatus and bloody contents of the bowels.

He attributed the cause of this strange disease to the second consignment of cotton-seed cake meal which he was then feeding ; it was coarser, of a different color and less pleasant to the smell than the first. The cattle did not take hold of it with relish. Some refused it altogether, a number of them took sick, some died and others recovered. He informed the superintendent of his suspicions, but the food was not changed until the death reports increased daily.

A superficial inspection of this food showed that it consisted of coarse crushed hulls, of a brown color, covered with downy fibrous substance, which at first sight looked like mold, and very likely mold was associated with it.

Whether this cattle actually succumbed to this cotton-seed cake meal is an open question. Still, in reviewing Dr. E. Pott's description regarding properties of this cake and meal in Vol. I, p. 428, of the Thierärztliche Encyclopedia, edited by Dr. Koch, Vienna, I notice an intimation which will admit of such a conjecture. After giving a brief classification of the different kinds of gossypium from which the seed is obtained, he says : "Upon the extraction of the oil, these cakes are of various qualities and not always fit for food.

"Cotton-seed cakes, meal, etc., are brought to market containing, besides the hard, indigestible ground hulls, a good deal of cotton fibres, and are, therefore, fit only for fertilizing. If utilized for food, they call forth violent disturbances in the digestive organs, and upon continuous feeding may eventually cause death, as the cotton fibres ball together in the alimentary track, thus producing constipation and inflammation of the bowels. Cakes made from hulled seeds are most nutritious, digestible and palatable. The better quality (unhulled) cakes are made from the Egyptian seed, it being easily freed from fibres ; it is pressed whole, and as an article of food is much sought after in England.

"Those cakes (unhulled) full of fibres are of a dark-brown color; and those containing less fibres are, on the other hand, of a greenish color, but soon turn brownish. Both these last named kinds, as a rule, contain, in addition to the hulls and fibres, other impurities, such as minute particles of iron from the presses, etc., and are often, as are all other cakes stored in damp places, impregnated with mold and other organisms, through which poisonous alkaloids (ptomaïnes) seem to form in the cakes to such a degree that their consumption may have a fatal effect on the cattle.

"The good quality of hulled cakes, the so-called American cotton-seed cakes, are of a bright yellow color; if they be of a dark color they have either been pressed while they were too warm, stored in a damp place, or spoilt in some other way, and therefore of doubtful quality. Well-prepared cakes should have an agreeable odor, a nut-like sweet taste, be hard and dry. Cakes and meal made from sound hulled seeds are eaten with relish and promote the thrift of all domestic animals used for draught and food.

"To milch cows is given daily three pounds, sometimes as high as five pounds; to draught oxen, three to four pounds; to oxen for fattening purposes, as much as six pounds; to sheep and swine, half to one pound; to horses, one to two pounds.

"Finally, the main inducement for bringing cotton-seed cake into widespread use is its cheapness."

